# Spontaneous facial expressivity predicts real-world social network size and richness

**DOI:** 10.1016/j.isci.2026.115972

**Published:** 2026-06-05

**Authors:** Eithne Kavanagh, Alisa Balabanova, Jasmine Rollings, Robin I.M. Dunbar, Jamie Whitehouse, Bridget M. Waller

**Affiliations:** 1 Department of Psychology, Nottingham Trent University, Nottingham, UK; 2 Department of Experimental Psychology, University of Oxford, Oxford, UK

**Keywords:** Psychology, Social interaction, Social sciences

## Abstract

Humans have highly expressive faces, yet from an evolutionary perspective, the adaptive benefit of such expressivity remains unclear. One possibility is that facial expressivity confers social benefits. Here, we provide empirical support for this idea by demonstrating associations between individual-level facial expressivity and social network metrics. We recorded spontaneous social interaction across five studies (*N* = 1039) and extracted quantitative expressivity indices using the automated facial action coding system. We analyzed expressivity in relation to real-world social networks. Increased expressivity predicted larger, more diverse, and more embedded social networks in women, but not men. However, expressivity predicted more one-to-one social relationships across genders. Although correlational, these findings could suggest that facial expressivity functions to foster more intimate social connections. This provides support for the hypothesis that the need for close interpersonal bonds has driven the evolution of high levels of facial expressivity in humans.

## Highlights

• Facially expressive women have richer real-world social networks
• Facially expressive men and women have more one-to-one social connections
• Facially expressive men and women are more likable in first impressions

## Graphical abstract

**Figure:**
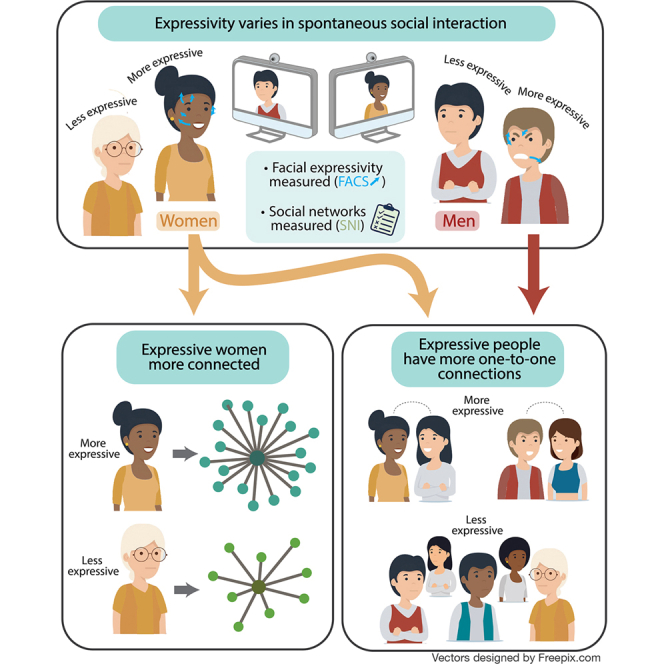

## Introduction

The human face is in near-constant motion during social interaction, with an approximate average of 100 facial movements per minute.^1^ It is capable of producing 46 distinct movements, which can form thousands of combinations.^2^^,^^3^ Indeed, an absence of facial movement is unusual enough in humans that an entire field of research examines infants’ responses to a “still face.”^4^ Among other mammalian species, humans’ capacity for facial expressivity is a clear outlier,^5^ and even other primate species appear to produce facial movement at a considerably lower rate.^6^^,^^7^ This suggests strong selection pressure on facial expressivity in human evolution. Despite this, evidence of its adaptive value is missing.

Converging evidence indicates that the tendency to be more or less facial expressive is an individual trait stable across contexts, time, and social partners.^1^^,^^8^^,^^9^^,^^10^ Facial expressivity varies considerably across individuals, a variability that appears to be much more limited in other primate species.^6^ The outcomes of this variation can be examined to unveil the function of facial expressivity as a behavioral phenotype, but behavioral variability is often dismissed as noise.^11^

In terms of immediate social outcomes, evidence suggests that facial expressivity increases affiliation.^12^ In first impressions, more facially expressive people are more liked by a social partner and third-party observers,^1^^,^^10^^,^^13^ and are more popular during group formation (78). While these data are suggestive of an adaptive advantage, it is not clear whether this proximate affiliative benefit results in ultimate fitness benefits. In order to evidence the adaptive value of facial expressivity, it is necessary to demonstrate broader fitness consequences outside of immediate affiliative outcomes, such as its relation to wider social connectedness.

An individual’s social network encompasses the full scope of other individuals with whom they maintain some degree of regular interaction.^14^ One’s social network is the single most robust predictor of a range of survival and reproductive benefits.^15^ Individuals with larger, more diverse social networks live longer lives, have better cognitive and physical function,^16^ are less susceptible to infectious illness^17^ have greater access to resources,^18^ and have greater reproductive success.^19^ Metrics of individual-level social network can therefore be considered a *de facto* fitness benefit.^15^

Along with exceptional levels of expressivity, humans also have exceptionally large and complex social networks.^20^ We propose, therefore, that extensive facial expressivity has evolved in humans by affording the building and maintenance of larger, more diverse social networks. Humans’ uniquely large and multi-level networks are a result of a range of structural, behavioral, and cognitive factors which bolster social bonding, cooperation, and conflict management, solving problems that constrain group size in other species.^15^ Evidence suggests that in other primate species (e.g., rhesus macaques), more facially expressive dominant males have more socially cohesive groups.^6^ Humans may have leveraged this function more than all other primates, however.^21^ While there is currently no direct evidence linking facial expressivity to social networks in humans, populations with atypical or infrequent facial expression often experience social difficulties (e.g., autism and Parkinson’s disease;^21^^,^^22^).

Beyond giving a positive first impression, facial expressivity could foster the longer-term development of social connections by enhancing one’s predictability. By signaling internal states and intentions, facial expressions allow others to predict future behavior.^23^ Being more predictable could improve social relationships by providing others with the opportunity to respond optimally to one’s intentions, build trust, and cooperate appropriately. Supporting this suggestion, more expressive people are easier to read^10^^,^^24^ and more readable people are preferred as social partners.^10^^,^^25^^,^^26^^,^^27^ Likewise, when combined with agreeableness, expressive people achieve better outcomes in conflict.^10^ Management of conflict is a crucial aspect of successful relationship maintenance^28^^,^^29^ and likely subject to strong selection pressure. Non-human primates predict more peaceful outcomes when they observe social interactions with facial expressions compared to neutral expressions,^30^ and more facially expressive rhesus macaques have lower conflict-related injury (80). Taken together, these findings suggest that facial expressivity may be a crucial mechanism for navigating moment-to-moment dynamic social connections, ultimately contributing to the maintenance and expansion of social networks.

Our hypothesis fits within theoretical frameworks that focus on adaptive function and does not assume facial behavior is limited to emotional expression. The functional role of facial expression and its fitness benefits are often ignored in the literature. We argue that this is a result of the dominant emotional framework, which positions facial behavior as “outpourings” of internal emotional states, the Basic Emotions Theory of Facial Expression.^31^; Historically, this has attracted focus on the producer of the facial expressions, disregarding the response of the receiver and thus the resulting social outcomes.^23^^,^^32^ These outcomes, however, are key to understanding why extensive facial expressivity has evolved in humans. This is not to say that the communication of emotions does not have social outcomes; indeed, emotional expressions can have strong effects on receivers.^33^

However, facial expression communicates more than emotion (e.g., punctuating speech, back-channeling; 34, 35) and evidence of a correspondence between facial expression production and emotional experience is weak.^34^ Alternative frameworks that decentralize the role of emotion in understanding facial expression are receiving increasing support. For instance, the Behavioral Ecology View of Facial Expression BECV^35^^,^^36^; proffers that facial expressions in conversation communicate social intentions, which may or may not be tied to emotion. We do not assume that emotions cannot be conveyed through facial behavior, or that they do not play a privileged role in social communication. We also do not aim to provide evidence in support of any theoretical perspective. Rather, we work under the assumptions of BECV; that facial expression extends beyond emotion and should be understood in terms of its evolved function and not just the internal state they represent.

### Current study

Here, we quantified real facial behavior from large-scale video datasets of participants engaging in social interaction (in-person, via video-call, dyadic, in a group, or solo but directed toward a perceived social partner). As recognized in a recent *Nature Human Behavior* feature article, the future of human behavior research demands improvements in ecological validity.^37^ The majority of the body of facial expression literature measures facial behavior via self-report questionnaires, e.g.,^38^ or relatively artificial non-social laboratory studies, e.g., posing facial expressions, reacting to video stimuli^39^^,^^40^ so the need for better ecological validity is particularly stark. This is likely due to the time-consuming nature of collecting naturalistic data and manually coding facial behavior. Recent technological advances, however, have made automated facial behavior coding of extensive video data possible, allowing for quantitative facial expressivity measures in a large sample of participants.

We examined how the size, diversity, and embeddedness of participants’ social networks varied as a function of their facial expressivity. These metrics reflect distinct qualities of an individual’s social network that can offer fitness benefits in different ways.^17^ The size reflects the total number of social connections a person has (i.e., people they interact with, with some degree of regularity). The diversity reflects the number of different types of social connections a person has (e.g., a friend, a parent, and romantic partner). The embeddedness reflects the number of domains (e.g., family) in which participants have many high-contact social connections. Together, they capture the richness of participants’ social network.

We incorporated participants’ gender, as there are distinct gender differences in the management of social networks. In comparison to men, women tend to have larger and more diffuse social networks,^41^ with multiple emotionally intense and supportive social connections.^42^^,^^43^ Women, on average, tend to prefer more intimate dyadic relations while men tend to prefer larger group-based relations.^44^ People also tend to have more same-gender than different-gender social connections in their networks.^45^ We explored whether facial expressivity plays a role in these gender differences in social network composition.

This is a correlational investigation, and so a causal relationship between facial expressivity and social network metrics cannot be inferred. However, our aim is to provide initial associative evidence for the adaptive function of facial expressivity, offering insight into why such high levels of facial complexity have evolved in humans.

## Results

### Relationship between facial expressivity and liking

Expressivity predicted being more liked by a new social partner, similarly in men and women [see data analysis section Table 1 for summary of models. Model 1; marginal R^2^ = 0.007, interaction dropped: ΔAIC = 0.44, expressivity: *t*(618) = 3.86, β = 0.04, 95% CI(0.02, 0.06), *p* < 0.001], which replicates and extends the findings of Kavanagh et al. (2024) by incorporating gender as an interaction with facial expressivity.

**Table 1:** Model formulas

Model	Outcome	Predictors
1	liking	∼ facial Expressivity ∗ Gender + Group Size + (1|Dataset/Group ID)
2	network size	∼
3	network diversity	∼
4	embedded network	∼
5	facial expressivity	∼ gender + group size + (1|dataset/group ID)
6	network size	∼ gender + (1|dataset)
7	network diversity	∼
8	embedded network	∼
9	N Female Ties	∼facial expressivity ∗ gender + N one-to-one ties + N males ties
10	N Male Ties	∼facial expressivity∗ gender + N one-to-one ties + N females ties
11	N One-to-one Ties	∼facial expressivity gender + N group ties + N females ties
12	N Group Ties	∼facial expressivity∗ gender + N one-to-one ties + N females ties

Note: Models 1 and 5 are linear mixed models, and models 2–4 and 6–8 are generalized linear mixed models. Models 9–12 are (non-mixed) generalized linear models.

### Relationship between facial expressivity score and social network measures

For the main analyses (models 2–4), there was a significant interaction between expressivity and gender in predicting social network size [model 2; marginal R^2^ = 0.051, interaction retained: ΔAIC = 3.10, expressivity∗gender: *z* = 2.263, *b* = −0.06, 95% CI(-0.11,-0.01), *p* = 0.024], network diversity [model 3; marginal R^2^ = 0.009, interaction retained: ΔAIC = 2.28, expressivity∗gender: *z* = −2.067, b = −0.05, 95% CI(-0.11,-0.00), *p* = 0.039] and embedded networks [model 4; marginal R^2^ = 0.014, interaction retained: ΔAIC = 3.579, expressivity∗gender: *z* = −2.333, *b* = −0.12, 95% CI(-0.022,-0.02), *p* = 0.020].

**Figure 1:**
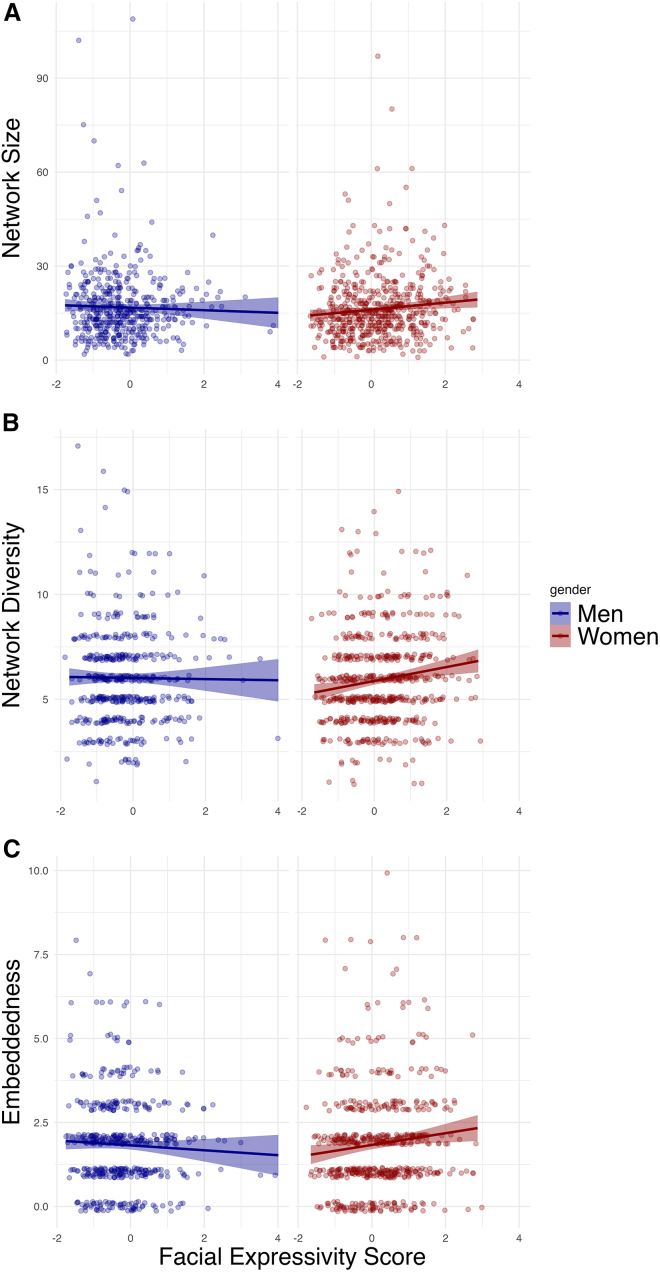
Relationship between facial expressivity score and social network measures, with color indicating participant gender (A) Relationship between facial expressivity score and network size. (B) Relationship between facial expressivity score and network diversity. (C) Relationship between facial expressivity score and embedded networks.

As illustrated in Figure 1, follow-up models indicated that in women, expressivity predicted network size [*z* = 2.785, *b* = 0.05 3, 95% CI(0.016,0.091), *p* = 0.005], network diversity [*z* = 3.920, *b* = 0.055, 95% CI(0.018,0.091), *p* = 0.003], and embedded networks: [*z* = 2.583, *b* = 0.091, 95% CI(0.022,0.160), *p* = 0.010]. However, expressivity did not predict any of the three social network metrics in men; network size [*z* = −0.491, b = −0.010, 95% CI(-0.050,0.030)], network diversity [*z* = −0.207, *b* = −0.004, 95% CI(-0.047,0.038), *p* = 0.836], or embedded network [*z* = −0.911, *b* = −0.038, 95% CI(-0.120,0.044), *p* = 0.363].

See SI1 [SI1A] for analyses including the three individual expressivity measures (rate, duration, and combination repertoire), which also predicted the three social network metrics in women (model statistics in SI [Table SI1A.1]).

### Gender comparisons in facial expressivity and social network measures

Women were significantly more facially expressive than men (model 5; marginal R^2^ = 0.049, gender: *t*(1004) = -7.228, β = −0.44, 95% CI(-0.560,-0.330), *p* <0 .001), yet contrary to predicted, there were no significant gender differences in any of the three social network measures; network size [model 6, marginal R^2^ = 0.002, gender: *z* = 1.458, β = 0.02, 95% CI (−0.01,0.05), *p* = 0.145], network diversity [model 7, marginal R^2^ = 0.000, gender: *z* = 0.425, β = 0.01, 95% CI (−0.04, 0.06), *p* = 0.671], and embedded networks [model 9, marginal R^2^ = 0.000, gender: *z* = −0.208, β = 0.00, 95% CI (−0.10, 0.08), *p* = 0.835]. This could point toward facial expressivity predicting only certain types of social ties that vary by gender, such as male and female social ties, or social ties that tend to be more dyadic (i.e., “one-to-one”) or group-based in nature.

### Relationship between facial expressivity and the number of social contacts

In a subset of the data (dataset 4; *N* = 297), facial expressivity did not specifically predict having more female social ties [model 9; Nagelkerke’s R^2^ = 0.828, interaction not retained: ΔAIC = 0.212, expressivity score: *z* = 0.403, *b* = 0.000, 95% CI(-0.03,0.05), *p* = 0.687) or male social ties [model 10; Nagelkerke’s R^2^ = 0.789, interaction not retained: ΔAIC = 0.808, expressivity score: *z* = 0.329, *b* = 0.00, 95% CI(-0.04,0.05), *p* = 0.742].

However, facial expressivity predicted having more one-to-one social ties regardless of participant gender [model 11; Nagelkerke’s R^2^ = 0.082, interaction not retained: ΔAIC = 0.808, expressivity score: *z* = 3.369, *b* = 0.08, 95% CI(0.04,0.13), *p* <0 .001]. Facial expressivity interacted with gender in predicting the number of group-based social ties [model 12; Nagelkerke’s R^2^ = 0.115, interaction retained: ΔAIC = 4.401, expressivity score ∗ gender: *z* = −2.516, *b* = −0.12, 95% CI(-0.22.,-0.03), *p* = 0.012). Post-hoc tests indicated that facial expressivity predicted more group-based social ties in women (*z* = 3.549, *b* = 0.118, 95% CI(0.052,0.183), *p* <0 .001) but not in men (*z* = −0.400, *b* = −0.017, 95% CI(-0.010,0.066), *p* = 0.689).

**Figure 2:**
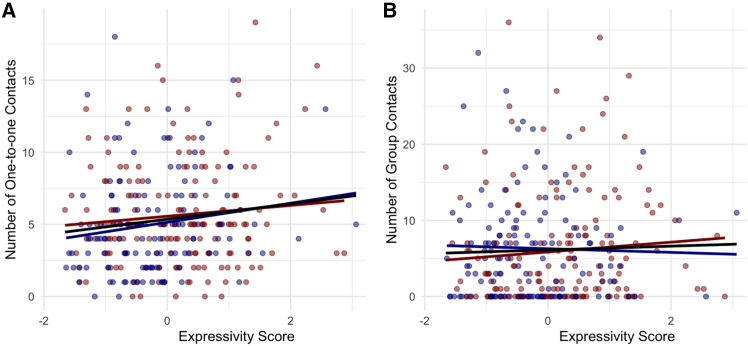
Relationship between facial expressivity and number of social contacts in men and women (A) Relationship between facial expressivity and the number of one-to-one social contacts. (B) Relationship between facial expressivity and number of group-based contacts.

These indicate that more facially expressive women have more one-to-one and more group-based relationships, while more expressive men have more one-to-one relationships only, as illustrated by Figure 2.

## Discussion

Here, we provide evidence of potential fitness benefits of facial expressivity as a behavioral phenotype in humans at the ultimate level. More facially expressive women had larger, more diverse, and more embedded social networks, while more expressive men did not. However, in a subsample, both facially expressive men and women had a higher quantity of one-to-one social ties than less expressive individuals, but only expressive women had comparatively more group-based social ties. Women were more facially expressive than men, but being more expressive was equally likable in initial first impressions in men and women. While future longitudinal or interventional studies are required to establish the direction of causality and to confirm temporal precedence, these findings provide initial evidence of an adaptive value of facial expressivity across genders, despite the stronger effect in women. Our findings suggest that high levels of facial expressivity may have been selected for in humans due to its role in expanding the richness of social networks and consequently improving survival and fitness.

The gender differences in our findings support the suggestion that facial expressivity has been a prime solution to barriers in group size over the course of human evolution (77). The “infertility trap” refers to a constraint on mammalian group size whereby the stress of group-living reduces fertility in females, thereby limiting group sizes to 15–20.^15^^,^^46^ Human social networks are well above 20 on average^20^; and according to our data. Dunbar^15^ outlines that one strategy to break this “glass ceiling” of social network size in a species is for females to buffer stress through forming close relationships with other females, or sometimes males. Our findings that women were more expressive than men, and that expressivity predicted more social relationships in women, point to facial expressivity as a possible mechanism for buffering the stress of group living through enhanced female social bonding. Higher facial expressivity could also have plausibly evolved in women to facilitate mother-infant interactions, providing an additional evolutionary pathway through which enhanced expressivity became advantageous, either initially or in parallel with broader social network functioning. This is an interesting avenue for future research. Alternatively, or in addition, there may be constraints on the effect of facial expressivity on social network metrics in men that affect women less so. Facial expressivity is most strongly predictive of one-to-one social ties. While facial expressivity provides an immediate affiliative benefit for men and women, in the long-term men report preferring to invest in group-based relationships^44^ where facial expressivity is apparently less beneficial. However, this research, and our findings, are based on the majority Western, Educated, Industrialized, Rich, and Democratic (WEIRD) samples, and gendered behavior varies cross-culturally.^47^ We therefore interpret any gender differences with caution.

Our hypotheses were based on facial expressivity enhancing social networks, and thus our interpretations of the findings are largely in line with this direction of causality. However, due to their correlational nature, we cannot rule out that having richer social networks increases facial expressivity, or that both are related to a third unmeasured variable. The increased social experience and confidence resulting from rich social networks might promote more uninhibited and engaging communication styles that increase trait-level facial expressivity over time. Such a causal direction is speculative, but empirical evidence points in the opposite direction; previous research indicates that facial behavior is a stronger predictor of affiliative outcomes than major variables in the literature associated with social benefits such as attractiveness and personality (78, 12). Nonetheless, an intervention study which modifies participants’ facial behavior, e.g., using radically open dialectical behavioural therapy^48^; and tests its long-term effect on social networks may be needed to confirm the direction of causality. It is plausible that a feedback loop exists, with expressivity enhancing social networks, and social networks enhancing expressivity, which is an exciting avenue for future research.

If indeed facial expressivity directly causes richer social networks and one-to-one contacts, this may occur at both the formation stage and maintenance stage of social relationships. We found facially expressive men and women to be more liked by a new social partner, potentially because being easier to read is socially appealing.^10^^,^^25^^,^^26^^,^^27^ In a longitudinal study, self- and observer-ratings of nonverbal expressivity predicted liking by social partners in first impressions and after nine-weeks of acquaintance,^49^ suggesting its impact at relationship formation and short-term maintenance. The facilitating role of facial expressivity in conflict management^6^^,^^10^ could impact the maintenance stage, as social partners who can read and predict each other are better able to navigate conflict without harming the relationship.^50^ Additionally, relationship decay is prevented by increased effort,^51^ and higher levels of facial expressivity could act as predictive signals of higher social motivation.^52^ These proposed mechanisms were not directly tested in the current research but should be tested in future studies.

The facilitating effect of facial expressivity on relationship maintenance may be particularly applicable to women and one-to-one relationships. Relationship decay is prevented more by conversation in women, but by doing activities together in men.^51^ Conversation is more likely to be facilitated by facial expressivity, given that it typically occurs face-to-face. Indeed, conversation might also be particularly effective in preventing decay in one-to-one relationships due to their more intimate nature. In comparison to group-based relationships, one-to-one relationships are likely to be more demanding in terms of moment-to-moment dynamic adjustment to the other’s social cues. As attention is comparatively more narrowly focused on the social partner,^53^ subtle facial communication can be attended to more readily. Similarly, failure to produce appropriate facial cues (e.g., backchanneling) could incur greater costs. Group-based contexts may benefit more from other communicative traits that are more salient in a group context, e.g., vocal communication.^54^ Interestingly, this would mirror Roberts et al.’s^55^ finding that chimpanzees use gestures (a relatively close-range communicative modality) more with close friends, yet use vocalizations more with distant group members. Regarding fitness benefits, the formation of dyadic differentiated bonds enhances reproductive success and longevity in social mammals.^56^ Thus, the link between facial expressivity and one-to-one relationships could reflect an adaptive social strategy.

The findings could be interpreted through an emotional lens. Emotional expressivity could enhance social networks via the social bonding effect of emotional sharing,^57^ perhaps particularly in one-to-one relationships. In support of this interpretation, evidence shows that greater positive emotional expression is associated with better social outcomes, while the suppression of emotions is associated with negative outcomes.^58^ However, negative emotional expression (particularly anger expression) also has negative or neutral outcomes,^58^ so on balance it is unclear whether the general tendency to be emotionally expressive would result in detectable social network benefits. This interpretation does not conflict with the behavioral ecology perspective, but rather represents how one aspect of facial communication (emotional expression) could contribute to the formation and maintenance of social relationships. It is likely that the facial behavior displayed by the participants included a mixture of displays tied to and not tied to emotional experience, both of which can be functional and influence relationship-building and position within social networks. Honest signaling of internal motivational or emotional states and socially strategic signaling may potentially operate at different explanatory levels but can jointly and dynamically shape social communication and contribute to social outcomes. Future work should explore the relative contribution of emotional and other communication to these benefits. Further, complementary approaches that combine behavioral data with neural or psychophysiological measures of social signaling and regulation could also help clarify how these processes interact across levels to produce the social outcomes observed. For instance, linking moment-to-moment facial behavior with psychophysiological measures (e.g., heart rate variability, skin conductance, EEG) could reveal how regulatory processes modulate facial signaling and its social consequences. This would facilitate situating the findings within a broader multilevel framework.

In conclusion, we have presented evidence that points to an adaptive, affiliative function of facial expression, and sheds light on the evolution of extensive expressivity in humans. Through the analysis of a large-scale quantitative dataset of facial behavior within real social interactions, this research contributes to addressing the lack of ecological validity in human behavior research.^37^ We advocate for the field moving away from self-report and subjective means of measuring facial behavior, and quantifying it directly. Our research also highlights the need to focus on individual differences in facial behavior, rather than the dominant focus on universality.^59^ This opens up potentially fruitful avenues for future research on the mechanisms linking facial expressivity to social networks, such as longitudinal studies tracking the path from first impressions to long-term social connections, and the determinants of individual variation in facial behavior. Future work could incorporate behavioral or partner-reported measures of predictability, conflict management, and interaction quality to test these mechanisms more directly.

### Limitations of the study

We acknowledge a number of limitations of this research, including the need to validate the findings cross-culturally and to ascertain the direction of causality. An evolutionary interpretation of our findings relies to some extent on their generalisability across differing populations. Members of individualistic cultures tend to endorse higher expressivity than those in more collectivist cultures,^60^ so the link between expressivity and social network benefits may be weaker or absent in collectivist cultures. Future research should address this concern. Additionally, a replication is needed to confirm our exploratory findings that facial expressivity predicts one-to-one social ties (and not group-based social ties) as these data were only available from a subset (*N* = 297) of participants. In two of the datasets, the video and social network data were collected months or years apart, which may have weakened observed associations due to temporal mismatch. Future research should investigate how expressivity and social networks co-vary dynamically over time. Moreover, although theoretically distinct, the three social network measures overlapped considerably and are egocentric. As such it is hard to tease apart how facial expressivity differentially relates to these dimensions at a system-level beyond individual pairwise associations. Social network analyses^61^ within self-contained networks of known individuals would allow such distinctions to be made. Finally, the exclusion of participants who identified outside of the gender binary (due to few cases in the dataset) means we cannot generalize our findings to this population.

## Resource availability

### Lead contact

Requests for further information and resources should be directed to and will be fulfilled by the lead contact, Eithne Kavanagh (eithne.kavanagh@ntu.ac.uk).

### Materials availability

This study did not generate new unique reagents.

### Data and code availability

• All anonymized original data have been deposited at Open Science Framework at https://osf.io/7hvjd and is publicly available as of the date of publication. Video data contains participants' personal images and is protected by GDPR and ethical guidelines and is not publicly available (with the exception of video dataset 4, which is publicly available upon request of the BetterUp team: https://www.betterup.com/research/candor-research). A subset of participants have provided optional consent for their video data to be shared with collaborators for use in future research. Requests to use the data in such a way in collaboration with the research team can be made by contacting the lead contact.
• All original code has been deposited at Open Science Framework at https://osf.io/7hvjd and is publicly available as of the date of publication.
• Any additional information required to reanalyze the data reported in this paper is available from the lead contact upon request.

## Acknowledgments

We thank research assistants Andrew Buckee, Olivia Keane, Natalia Ptaszynska, Barbara Devaney, Harry McCann, and Sundara Kashyap Vadapalli who supported data collection in datasets 2 and 5. We thank BetterUp, Andrew Reece, and the co-creators of the CANDOR dataset for providing the video data used in dataset 4. This project received funding from the 10.13039/501100000781European Research Council (ERC) under Horizon 2020 Research and Innovation Programme (grant agreement no. 864694) awarded to BW.

## Author contributions

Conceptualization: E.K., B.W., and R.D. methodology: E.K., B.W., J.R., A.B., and R.D. investigation: E.K., B.W., J.R., and A.B. visualization: E.K. and J.W. funding acquisition: BW. project administration: E.K. and B.W. supervision: B.W. writing – original draft: E.K. Writing – review and editing: E.K., B.W., and J.W.

## Declaration of interests

The authors have no competing interests.

## STAR★Methods

### Key resources table

**Table:** 

REAGENT or RESOURCE	SOURCE	IDENTIFIER
**Deposited data**		

Original data deposited for this study	Open Science Framework	https://osf.io/2ygcx/
CANDOR open-source videos (used in Dataset 4)	Better Up Website	https://www.betterup.com/research/candor-research

**Software and algorithms**		

Automated software to code facial movement	iMotions Facial Coding software	https://imotions.com/
Facial Action Coding System	Ekman et al.^2^	https://www.paulekman.com/facial-action-coding-system/
Survey software to collect self-report data	Qualtrics	https://www.qualtrics.com/
*lmerTest*	Kuznetsova et al.^62^	https://cran.r-project.org/web/packages/lmerTest/
*lme4*	Bates et al.^63^	https://cran.r-project.org/web/packages/lme4/
*performance*	Lüdecke et al.^64^	https://cran.r-project.org/web/packages/performance/
*effectsize*	Ben-Shachar et al.^65^	https://cran.r-project.org/web/packages/effectsize/
*emmeans*	Lenth et al.^66^	https://cran.r-project.org/web/packages/emmeans/

**Other**		

Gorilla experimental tasks (used in Dataset 5)	Gorilla Open Materials	https://app.gorilla.sc/openmaterials/1032571
Social Network Index Questionnaire (with modernisations; see SI [SI3])	Cohen et al.,^17^	https://db.arabpsychology.com/scales/social-network-index-sni-2/

### Experimental model and study participant details

We combined datasets from five studies with a total of 1039 participants (474 male, 565 female; see below Table), aged 18–91, all fluent English speakers. Full details of the samples and procedures for datasets 1–3 can be found in their accompanying publication (below Table). Dataset 4 includes a subset of participants from a previous study in which facial expressivity of participants was extracted from videoed dyadic interactions.^10^^,^^67^ This subset participated in a follow-up study to provide social network data (on Qualtrics via Prolific, £8 compensation, age 21–67). Participants from dataset 5 (aged 19–77) were recruited via Prolific (www.prolific.com), compensated £9 and completed the study on Gorilla (www.gorilla.sc). Further details about procedures for datasets 4 and 5 are provided in SI2. This includes details of country of origin, but details on the ancestry, race, ethnicity, and socioeconomic status of participants was unavailable.

**Table 2:** Summary of five datasets

Dataset	Total N	N Males	N Females	Video social interaction	Accompanying publication
1	31	15	16	• dyadic interaction with confederate • online • 2 min	Kavanagh et al.,^10^; Study 1, Neutral Context
2	65	16	49	• 3 x dyadic interactions with 3 x confederates • one in-person, two online • 3 min each	Rollings et al.^1^
3	254	133	121	• group interaction with 2–3 other participants • online • 5 min	Balabanova et al., in review
4	297	126	171	• dyadic social interactions with other participants (mean 2.42 SE 1.61 interactions per participant) • online • first 5 min analyzed	Kavanagh et al.,^10^; Study 2 Reece et al.,^67^
5	392	184	208	• 3 x solo videos, directed to a perceived social partner. • online • Video S1 (Hobbies): 30 s Video S2 (Scripted): mean 37.57 SE 7.75 s). Video S3 (Participant-directed): 30 s	Methods section of current paper
Total	1039	474	565		

The final sample excludes participants whose video data quality was inadequate (facial landmarks not reliably detected in more than 10% of video frames according to the facial coding software).

Participants whose gender was unavailable or outside of the gender binary were too few for meaningful comparison (*N* = 15). We therefore excluded these participants as gender comparison was a key focus of the study, and we also did not attempt exploratory analyses with these data.

This research received approval from NTU BLSS ethics boards (Application IDs; Dataset 1: ID WALLER 2023/262, Dataset 2: ROLLINGS 2022/126, Dataset 3: 2022/119, Datasets 4 and 5: 1767158). Informed consent was received from all participants.

### Method details

#### Video data

All datasets included video data of participants’ facial behavior directed toward a social partner (while unaware facial behavior was the primary focus), amounting to 6069 min (over 9 million frames) of codable video footage. In datasets 1–4 participants engaged in unstructured, free-flowing social interactions with another participant or a confederate, either in-person or online (see above table for further details; see below figure for video examples).

**Figure 3:**
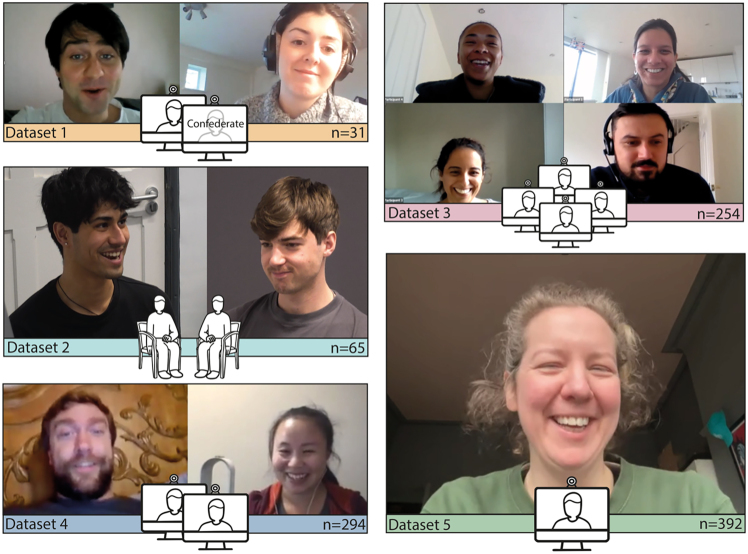
Still image exemplars of video social interaction data from each of the 5 datasets showing the range of settings

In dataset 5 participants were video recorded in three solo tasks (these tasks are openly available for replication on Gorilla open materials, including quality control instructions https://app.gorilla.sc/openmaterials/1032571). In Video S1, they spoke for 30 s about their hobbies. In Video S2, they read a predetermined script, designed to last approximately 30 s with a balance of positive, negative and neutral valence. In both videos, they were instructed to speak naturally as though meeting a new friend. In Video S3, they spoke freely for 30 s to another participant they were (falsely) informed they were paired with. They were told the other participant will view their video and decide how to allocate a bonus reward. Participants could not view themselves on-screen during the recordings, but could do so beforehand to test their facial visibility. See SI [SI2] for more detailed video instructions and electronic supplementary materials for example video clips. We validated the solo videos by comparing them with dyadic social interactions in three datasets (*N* = 249 participants; see SI [SI1B]). Facial expressivity during dyadic social interactions was significantly correlated with expressivity in all of the short video types in every dataset, with correlation coefficients ranging from *r* = 0.33 to *r* = 0.71. This indicates the short videos were valid proxies of expressivity within social interaction with others. This aligns with the concept of “thin slices of behavior”, where even brief excerpts of behavior can reliably reflect typical trait-like tendencies.^68^ Facial expressivity was also highly correlated across the three short videos (see SI [SI1B]).

Video S1. Exemplar of hobbies clip from dataset 5

Video S2. Exemplar of partner-directed clip from dataset 5

Video S3. Exemplar of scripted clip from dataset 5

The variation in methodologies across datasets should have minimal impact on testing our predictions, and we demonstrate in SI [Figure SI1C.2] that the slopes of the main analyses are similar when separating by context (solo, dyadic, group), modality (online, in-person/online), and task structure (unstructured social interaction, semi-structured tasks) to the overall model slopes. Converging evidence indicates that facial expressivity is a stable trait demonstrating within-individual consistency across behavioral contexts, social partners and time,^8^^,^^10^ This is further supported by expressivity in the above short clips corresponding with that in longer dyadic interactions. While absolute levels of expressivity may differ to some degree across contexts such as online vs. in-person, the predictive ability of expressivity on immediate affiliative outcomes does not differ.^1^ Testing our predictions should also be unaffected by the use of confederates in some datasets. The included interactions were unstructured without experimental manipulation, making confederate-participant interactions essentially equivalent to those of any dyad. Importantly, affiliative outcomes of expressivity are also similar whether judged by a confederate, another participant, or a third-party rater.^10^ As such, using various approaches to capture a snapshot of an individual’s trait level of expressivity and controlling for dataset in statistical models is appropriate for testing our predictions.

#### Measures

##### Facial expressivity

Facial behavior was coded from the videos using automated software iMotions^69^ based on Facial Action Coding System FACS.^2^; FACS is the gold standard in facial behavior measurement, as it is an objective and quantitative approach that avoids subjective facial expression categorisation. It uses “action units” (AU) as units of measurements which refer to individual facial movements based on underlying musculature. Using Emotient’s FACET technology (www.imotions.com/emotient), iMotions allows for extraction of sixteen AUs (we excluded AU25, AU26 and AU43 and “smirk” as they cannot be meaningfully differentiated from speaking, blinking or asymmetrical AU12).

From these, we first extracted six facial expressivity measures. A principle components analysis (PCA) on these measures indicated the two component structure found in Kavanagh et al., (2024) was suitable (see SI [SI1D], PCA results in Table SI1D, correlation matrix of measure in Figure SI1D). We focused on the first component, comprised of the rate, duration and combination repertoire of action units (below Table) as Kavanagh et al., (2024) found this component to be implicated in social affiliation (analyses with component 2 are available in SI [SI1F]; model statistics in Table SI1A2). As such, we calculated a single facial expressivity score per participant from the mean of these three measures (standardised within each dataset, to account for dataset differences such as length of interactions; unstandardised values for each dataset are provided in SI [SI1C], illustrated in Figure SI1C.1). In datasets with more than one interaction (datasets 2, 4, and 5), an expressivity score was calculated per interaction, and the mean score across all interactions was used per individual in all analyses.

**Table 3:** Facial expressivity measures and their calculations

Expressivity Measure	Calculation
Rate	the N of AUs produced per minute
Duration	the percentage time each AU was produced, summed.
Combination Repertoire	the total N of unique AU combinations (i.e., simultaneous production of 2 or more AUs).
Expressivity Score	the mean of the Z-scores of rate, duration and combination repertoire

##### Liking

In datasets 1–4, participants’ social partners rated them on how much they liked them on a continuous scale. The liking ratings were standardised within each dataset as they were reported on different scale sizes.

#### Social network indices

Participants completed Cohen et al.’s^17^ established and highly-cited self-report Social Network Index (SNI). This assesses the extent and diversity of social ties across multiple domains, and has been found to reliably predict several health and wellbeing outcomes e.g., emotional life quality,^70^; common cold resistance.^71^; Self-report is a suitable approach to quantify social networks as it closely aligns with actual social contact,^72^ although memory constraints and variation in participant effort often limit the comprehensiveness of social network capture in methods such as free recall.^73^ The SNI limits these issues by prompting responses across a wide range of predefined social contexts (e.g., work, school) and relationships (e.g., parents, friends). Participants also indicate which social ties they communicate with at least once in a two-week period, which are classed as high-contact ties. We included some modifications of the original questionnaire to modernise it (e.g., including instant messaging; see SI [SI3]) and to maximise the likelihood of capturing participants’ entire social network. The video and SNI data were collected together in datasets 2–4 but in datasets 1 and 2 the SNI data was collected in a follow-up study up to ∼7 months (dataset 1) and ∼4 years (dataset 5) after the video data were collected. This timepoint difference should minimally affect testing our predictions given the trait stability of expressivity (beyond adding statistical noise), and excluding these datasets did not alter significance of models (see SI [Table SI1E]).

From participants’ responses, three social network indices were derived:

**Social Network Size:** The total number of high-contact social ties.

**Social Network Diversity:** The number of different social roles; i.e., domain (e.g., romantic relationship, work) in which the participant has at least one high-contact social tie.

**Embedded Networks:** The number of distinct network domains in which the participant is active. To count as an embedded network, the participant must have at least four high-contact ties within that domain. For the family domain, this requires at least three family social roles and four high-contact social ties.

These three metrics are closely intercorrelated (see SI [Figure SI1F]), but we retain them separately in keeping with the broader literature using these measures as they represent theoretically distinct aspects of social network as a multidimensional construct e.g.,.^74^^,^^75^

#### Social network composition/relationship types

To explore the gender differences in the link between facial expressivity and social network measures, we collected additional data in dataset 4 on participants’ social network composition. Previous literature indicates gender differences in the composition of social networks. Some evidence suggests that social ties may tend toward gender homophily,^45^^,^^76^ and women tend to favor dyadic relationships while men typically prefer group contexts.^44^^,^^77^ It is possible that facial expressivity may not benefit only women, but rather could foster the types of relationships (i.e., one-to-one, female) they tend to prefer. Therefore, we asked participants to indicate the gender of all high-contact social ties, and whether they typically spend time with each person one-to-one or in a group context using a five-point Likert scale. Social ties marked as “Mostly” or “Always” one-to-one were categorised as “one-to-one contacts”, and those marked as “Mostly” or “Always” in group context were categorised as “group contacts”. Those indicated as “equally one-to-one and in group” were not analyzed further.

### Quantification and statistical analysis

All analyses were conducted in R studio^78^; see Table 1 for model formulas.

To test whether liking and social network metrics were predicted by expressivity as a function of gender (models 1–4), we constructed mixed models including random factors of group ID (to account for participants in dataset 3 interacting in a group with other participants, treating each solo or dyadic interaction from other datasets as a unique level/group) nested within dataset (as each dataset contains its own distinct groups, with no overlap between datasets). We controlled for group size (solo videos = 1, dyads = 2, groups of 3 = 3, groups of 4 = 4). We compared AIC values for models with and without the interaction term and retained the interaction only when it improved model fit, defined as a reduction in AIC of at least 2.^79^

To test gender differences in expressivity and social network measures (models 5–8), we constructed four additional mixed models. We did not include group size or group ID in models 6–8 as group composition is unrelated to any outcome or predictor variable.

To test whether different types of social ties were predicted by expressivity as a function of gender, we constructed generalised linear models (models 9–12) using data from dataset 4 only. We controlled for other types of social ties found to be significantly related to expressivity in other models.

In models with liking and facial expressivity as outcome measures (models 1 and 5), we fit linear mixed models using *lmer* function in package *lmerTest*.^62^ Degrees of freedom were estimated using the Kenward–Roger approximation. In models with social network metrics as outcome variables we fit generalised linear mixed models (models 2–4, 6–8) using the *glmer* function in package *lme4*,^63^ specifying a Poisson distribution with a log link, as these variables are count data and this distribution provides the best fit. Inferences for fixed effects used Wald z-tests, which rely on asymptotic approximations. Generalised linear models were fit using *glm* function in base R, specifying a Poisson distribution with a log link. Effect sizes were calculated using function *r2* in package *performance*^64^(random-effects terms with zero or near-zero variance were removed to avoid singular model fits) and *standardize_parameters* in package *effectsize*^65^*.* Post-hoc tests were conducted using the *emtrends* function in package *emmeans*.^66^

The final models include only complete data, as datasets were excluded from a model if the required variables were not collected. Individuals with insufficient facial data quality, or without male/female gender identity, were excluded. Within each dataset, all other variables that were collected were available for all participants. All datasets include data on facial expressivity, the three SNI measures, and gender identity. Liking data were not collected in dataset 5, and the numbers of male, female, one-to-one, and group ties were only collected in dataset 4.

## Supplemental information

Document S1. Figure S1, Tables S1 and S2, Methods S1–S3, and supplemental references
