# Supplementary material for: Spontaneous facial expressivity predicts real-world social network size and richness
Source: iScience. 2026 Jun 5;29(6):115972. doi: 10.1016/j.isci.2026.115972 (PMC13253098; doi:10.1016/j.isci.2026.115972)
Supplement: Document S1. Figure S1, Tables S1 and S2, Methods S1–S3, and supplemental references [file mmc1.pdf]

iScience, Volume *29*

Supplemental information

Spontaneous facial expressivity predicts real-world social network size and richness

Eithne Kavanagh, Alisa Balabanova, Jasmine Rollings, Robin I.M. Dunbar, Jamie Whitehouse, and Bridget M. Waller

Supplemental information

Methods SI1: Additional Analyses

a) Main Analyses with Individual Expressivity Measures and Expressivity Component 2

Table SIA.1 provides the result statistics for nine generalised linear mixed models testing the relationship between the individual expressivity measures as predictors of social network metrics in women. These show that all expressivity measures significantly predict the three social network metrics with the exception that combination repertoire does not significantly predict network diversity. AIC values indicate that of the three expressivity measures, combination repertoire most strongly predicts network size, rate most strongly predicts network diversity, and duration most strongly predicts embedded network.

We also conducted the three main analyses examining the relationship between expressivity and social networks metrics using component 2 from the PCA (see SI1D), which is comprised of AU repertoire, corrected AU repertoire and diversity score. We used the same approach as with expressivity component 1 score (see main text models 2-4).

Results (see table S1A.2) indicated a significant main effect of expressivity score 2, but no interaction between expressivity score 2 and gender, on network size and embedded network. There was a significant interaction between expressivity score 2 and gender on network diversity. Post-hoc tests indicate that expressivity score 2 predicted network diversity for women but not men.

Table S1A.1

Generalised linear mixed models with social network metrics as outcome variables and individual expressivity measures as predictors, in female participants only. All models fitted with a poisson distribution and log link. All predictors are standardised to correct issues of convergence. Related to Figure 1

Outcome	Predictor	B	SE	*z*	*p*	AIC
Network Size	Rate	0.051	0.011	4.888	<.001***	5381.569
	Duration	0.056	0.011	4.934	<.001***	5382.565
	Combination Repertoire	0.084	0.013	6.399	<.001***	5364.867
Network Diversity	Rate	0.044	0.017	2.562	.010*	2448.627
	Duration	0.038	0.019	2.008	.045*	2451.409
	Combination Repertoire	0.042	0.022	1.906	.057	2452.138
Embedded Network	Rate	0.070	0.031	2.279	.023*	1928.965
	Duration	0.084	0.031	2.694	.007**	1927.085
	Combination Repertoire	0.071	0.030	2.399	.017*	1929.646

Note: * *p*<.001, ** *p*<.01, ** *p*<.05

Table S1A.2

Results from main analyses (generalised linear mixed effect models 2-4) including expressivity score 2. Related to Figure 1.

Outcome Measure	Predictors	*z*	*p*
Network Size	Expressivity Score 2	2.764	.006**
	Expressivity Score 2 × Gender	-0.095	0.924

Outcome Measure	Predictors	*z*	*p*
Network Diversity	Expressivity Score 2 × Gender	-2.030	0.042*
Network Diversity (Females)	Expressivity Score 2	2.739	0.006**
Network Diversity (Males)	Expressivity Score 2	-0.118	0.906
Embedded Network	Expressivity Score 2	2.116	0.034*
	Expressivity Score 2 × Gender	-1.278	0.201

b) Short solo video clips comparisons with real dyadic social interaction

In three datasets (datasets 1, 2 and 4) we have facial expressivity data from the same participants in a real dyadic social interaction and in uploaded video clips. We therefore tested whether expressivity was similar in the clips compared to their real social interactions, as a means of validating the use of the clips as proxies for real social interaction. We have the ‘hobbies’ video clips across all three of these datasets, but the ‘scripted’ and ‘partner-directed’ video clips were only available from dataset 4.

Combining the three datasets (N=249), we observed a significant moderate correlation between facial expressivity in the real social interaction and in the hobbies clip [$r(243)= .44$, $p<.001$][1]. Examining the datasets individually, this correlation was strongest in dataset 2 [$r(45)=.71$, $p<.001$], followed by dataset 1 [$r(30)=.63$, $p<.001$] and then dataset 4 [$r(164)=.33$, $p<.001$]. Notably, the length of time between the social interactions and the video clips also ascends in this order; they were taken on the same day in dataset 2, ~7 months apart in dataset 1, and ~4 years apart in dataset 4. These data suggest that the hobbies video clips are a very good proxy for one’s level of expressivity within real social interaction on a given day.

The three video clip types (hobbies, scripted, partner-directed, available in dataset 4) showed strong correlations with each other [hobbies and partner-directed: $r(127)=.75$, $p<.001$, scripted and partner-directed: $r(125)=.72$, $p<.001$, hobbies and scripted: $r(130)=.72$, $p<.001$]. The mean expressivity across the three video clips showed a significant moderate correlation with expressivity in real social interaction [$r(124)=.40$, $p<.001$]. In comparison, correlations were slightly smaller using the scripted clip [$r(134)=.343$, $p<.001$], the partner-directed clip [$r(128)=.35$, $p<.001$] and the hobbies clip alone. These suggest that using three clips provides a somewhat stronger proxy for one’s expressivity in real social interaction than one clip type.

Together, these findings suggest that short video clips are a valid proxy for facial expressivity in social interaction, and three clips are better than one.

[1]We calculated Pearson’s R correlations across all analyses to facilitate comparison, although this resulted in the first analysis (hobbies and real interaction) suffering from pseudreplication due to pooling data from three datasets. However a linear mixed model with a random effect of dataset also indicated that expressivity in the hobbies clip significantly predicted expressivity in the real interaction [$F(1,243)=59.90$, $p<.001$)].

c) Dataset comparisons in facial expressivity

Figure S1C.1 illustrates the median and spread of unstandardised expressivity measures across the five datasets, colour-coded for the associated length of the video clips. Visually, this shows some differences across the datasets, with combination repertoire appearing to vary more than other measures as a function of video clip length. Figure S1E.2 illustrates the associative patterns of the main findings (expressivity score and the three social network metrics) in female participants according to different characteristics of the datasets; context (solo, dyadic, group), modality (online, mixed in-person and online) and task structure (unstructured social interaction, semi-structured tasks). Across categories, slopes are generally similar in magnitude and in the predicted direction, indicating that the main findings are robust to methodological variation across datasets. One exception is observed for the association between expressivity and embedded network in the mixed online–in-person category, which shows a negative slope. We interpret this result with caution given the small sample size in this category (N = 65) and the consistently positive slopes observed in all other categories.

Figure SI1C.1

Comparison of unstandardised facial expressivity measures across datasets. Related to table 2.

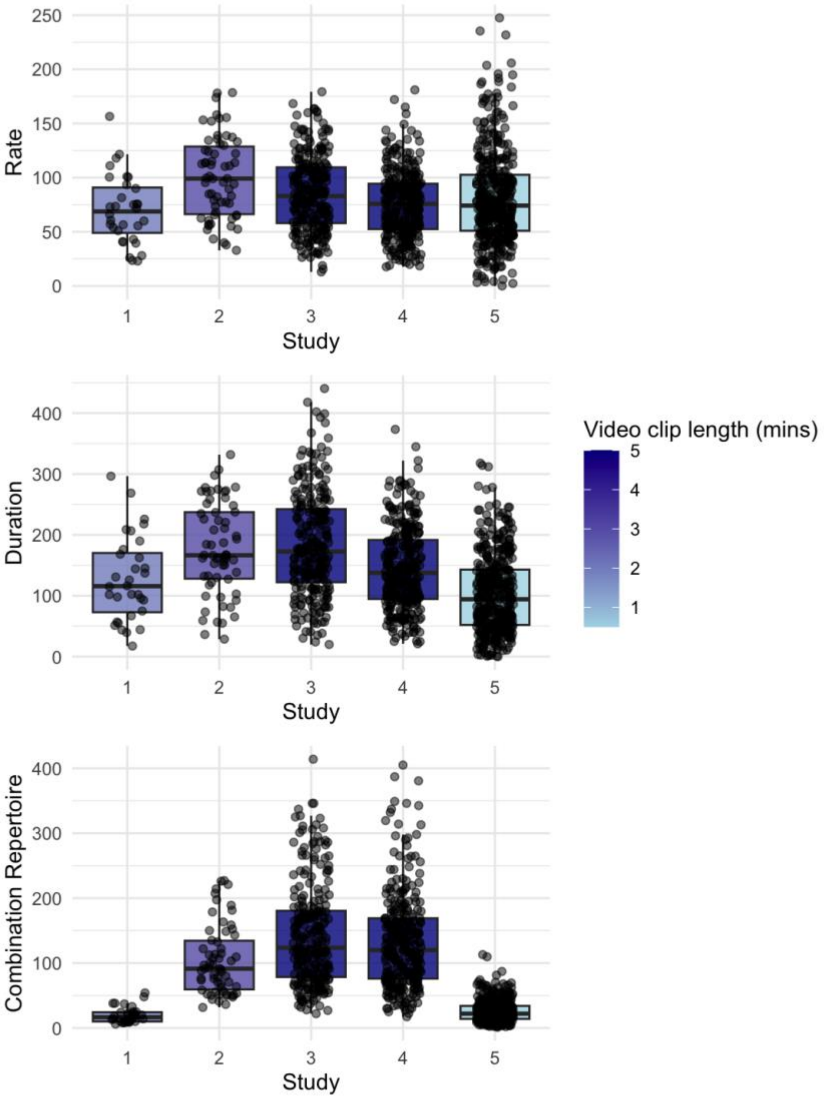

* Note: A darker shade of blue indicates a longer associated video clip length (Study 1=2minutes, Study 2=3 minutes, Study 3=5 minutes, Study 4=5 minutes, Study 5=30 seconds). Multiple clips were used to calculate expressivity for studies 2, 4, 5, but as the mean values across video clips was used per participant, the length of the individual clip was coded here.

Figure SI1C.2

Relationships between expressivity scores and social networks in female participants according to different characteristics of the dataset (context, modality and task structure). Related to figure 1.

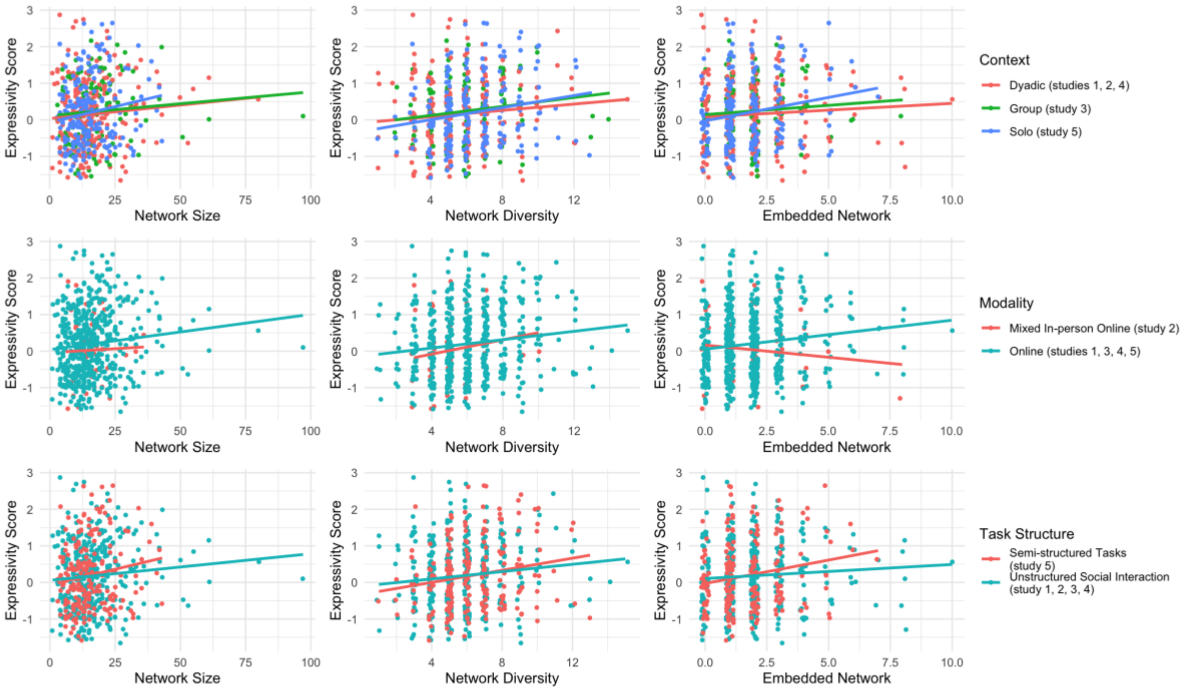

d) Principal Components Analysis

To examine whether the same 2-component structure identified in Kavanagh et al., (1; study 2) replicated in our sample, we conducted a principal components analysis (PCA) using the *psych* package (2). As the participants from dataset 4 in our sample were also in the analysis in Kavanagh et al., (1) we excluded their data from our analysis, resulting in a total sample size of 740 participants. We included the six facial expressivity measures (rate, duration, AU repertoire, corrected AU repertoire, combination repertoire and diversity score. A correlation matrix of these measures is provided in figure S1D. Bartlett's test of sphericity was significant (χ^2 (5)= 14849, p<.001), indicating it was appropriate to conduct PCAs. We conducted a PCA using an orthogonal varimax rotation and 2-component structure, and results indicated this was suited to the data (see table S1D). The root mean square of residuals was low (0.07), indicating a good fit of the model to the data. The variance explained by both components was similar (Component 1: 53%, Component 2: 47%). As in Kavanagh et al., (2024), rate, duration and combination repertoire loaded most strongly onto component 1, while repertoire, corrected repertoire and diversity score loaded most strongly onto component 2. Communalities for most variables are high, indicating that the two components explained a large proportion of the variance, while uniqueness values are low, indicating little variance was unexplained. Overall, the results indicated that it is appropriate to use the same two components using the same measures as in Kavanagh et al., (2024).

Figure SI1D

*Correlation matrix with six facial expressivity measures. Values indicate Pearson's R. *= p<.05, **= p<.01, ***= p<.001. Related to table 2.*

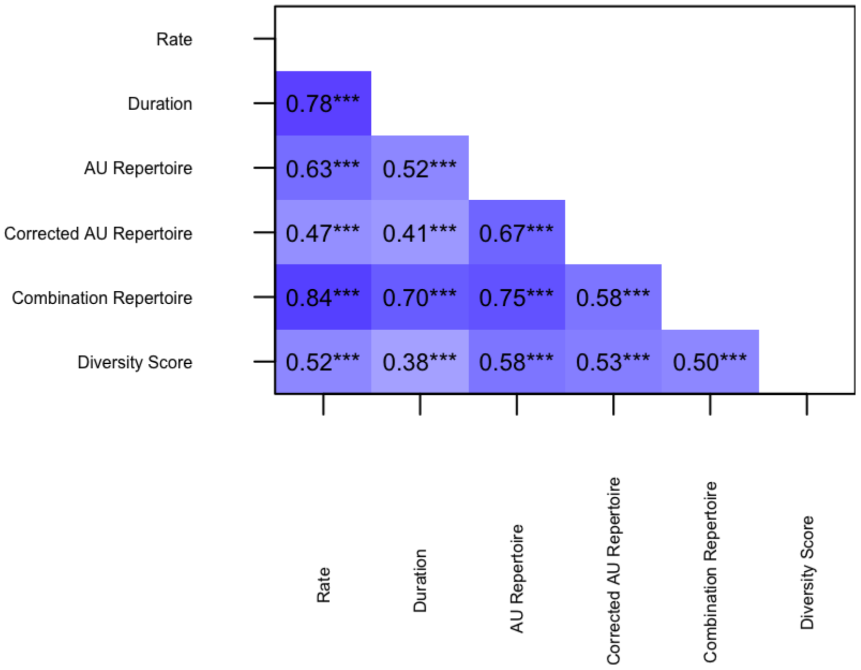

Table SI1D

Results from Principal Components Analysis forcing two component solution. Related to table 2.

Variable	Component 1	Component 2	Communality (h²)	Uniqueness (u²)	Item Complexity
Rate	0.88	0.34	0.89	0.11	1.3
Duration	0.90	0.17	0.85	0.15	1.1
AU Repertoire	0.49	0.74	0.78	0.22	1.7
Corrected AU Repertoire	0.24	0.83	0.75	0.25	1.2
Combination Repertoire	0.79	0.48	0.86	0.14	1.7
Diversity Score	0.22	0.88	0.69	0.31	1.1

e) Main Analyses Excluding Datasets 1 and 4

We conducted the three main analyses examining the relationship between expressivity and social networks metrics, including only those datasets in which expressivity and social network data were collected during the same testing session (datasets 2, 3 and 5). We used the same approach as those with the full sample (see main text models 2-4) and conducted post-hoc tests where there was a significant interaction. These models produced the same pattern of results as with the full sample (see table S1E).

Table SI1E

Results from main analyses (generalised linear mixed effect models 2-4) excluding datasets 1 and 4. Related to figure 1.

Outcome Measure	Predictor	z	p
Network Size	Expressivity Score × Gender	-2.527	.012*
Network Size (Females)	Expressivity Score	2.663	.007**
Network Size (Males)	Expressivity Score	-0.945	.345
Network Diversity	Expressivity Score × Gender	-2.164	.030*
Network Diversity (Females)	Expressivity Score	2.786	.005**
Network Diversity (Males)	Expressivity Score	-0.403	.687
Embedded Networks	Expressivity Score × Gender	-2.247	.025*
Embedded Networks (Females)	Expressivity Score	1.964	.049*
Embedded Networks (Males)	Expressivity Score	-1.267	.205

f) Social Network Inter-correlations

As illustrated in figure SI1, the three social network metrics showed moderate to high correlations (pooled across all participants).

Figure SI1F

*Pearson's R correlations between the three social network metrics, pooling all participants ***p<.001. Related to table 3.*

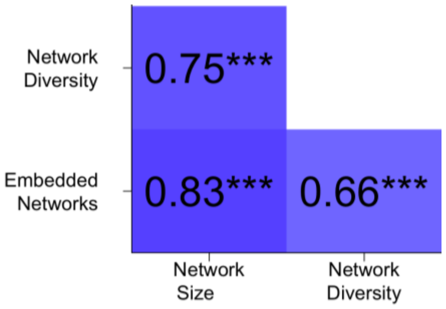

Methods SI2 Additional Study Details

In both dataset 4 and 5, participants were told they would be taking part in a study on social behaviour and relationships in online social interactions and would complete various video tasks and questionnaires. Participants completed three video tasks outlined below.

Video Tasks:

The three video tasks (hobbies, scripted, partner-directed) are available to preview and to re-use in future research from Gorilla open materials via the following link: https://app.gorilla.sc/openmaterials/1032571. This provides the instructions and displays participants were asked to follow for each video tasks (the 'Camera Visibility' task was completed before each of the three video tasks). The exact script participants were asked to read for the scripted video task was as follows:

Hello, my name is Alex. I work in an office Monday to Friday. It can be a bit boring, but it's close to my house so I don't have to travel far each day. After work I really enjoy going out to eat at nice restaurants with friends, or cooking tasty meals at home. I'm learning the guitar at the moment, so I practise playing at the weekend. My neighbours complained that I was too loud though, which is a bit embarrassing. I play in the garage now so they can't hear. I'm getting much better!

Exemplars of the three video clips are available to view in the electronic supplementary materials:

Video S2A : *Hobbies clip.* An example of a participant's uploaded 'Hobbies clip' in which they were instructed to speak about their hobbies for 30 seconds.

Video S2B: *Partner-directed clip.* An example of a participant's uploaded 'Partner-directed clip' in which they were instructed to talk with another participant they were told they matched with for 30 seconds.

Video S2C: *Scripted clip.* An example of a participant's uploaded 'Scripted clip' in which they were instructed to read out a script.

Dataset 4 Sample and Procedure

A sample of 1456 participants (all United States residents, recruited via online participant recruitment website Prolific; https://www.prolific.com/) took part in a recorded video call interaction with another participants as part of the CANDOR study in 2020 (3), of which 1315 had sufficiently valid facial expressivity data (1). We made a follow-up study available to these participants via Prolific and 297 completed this between September-October 2024 on Gorilla (https://app.gorilla.sc/; with the exception of the Emotional Recognition Index task which was obtained from the Swiss Centre for Affective Science https://www.unige.ch/cisa/emotional-competence/home/research-tools/eri/ available on Qualtrics; https://www.qualtrics.com/). Participation took approximately 30 minutes, and consisted of first reading participant information and providing consent, then completing the Social Network Index (SNI; 4) and then completing a battery of cognitive and social tasks presented in a randomised order. The SNI was completed before other tasks/questionnaires to prioritise its completion (accounting for the potential for partial completions). The tasks and questionnaires were chosen to address multiple research questions; the predictions outlined in the current manuscript, and other unrelated questions.

Questionnaires and Tasks (presented after the SNI in a randomised order)

- Video 1 – Hobbies (see video tasks section above)
- Video 2 – Scripted (see video tasks section above)
- Video 3 – Partner-directed (see video tasks section above)
- Berkey Emotional Expressivity Scale; Impulse strength subscale (5)
- Social Face Test: a novel task in which naturalistic social interaction video clips are shown and participants are asked to interpret the actors' intentions.

- Facial Expressivity Scale: a novel 9-item self-report questionnaire measuring facial expressivity
- Social Niche Scale: a brief (8-item) novel questionnaire designed to measure preference for and time spent in cooperative and competitive social environments.
- Emotional Recognition Index – Facial Expression Task only (6)
- Facial Production Task: a novel task in which participant are shown moving images of facial movements and asked to produce them while being video recorded.

Dataset 5 Sample and Procedure

Three-hundred and ninety-two participants were recruited via Prolific (See table S2; 28 % United Kingdom, 25% South Africa, 36 % Other European, 11% Other) between October 2023-January 2024. They completed the study on Gorilla (with the exception of the Emotional Recognition Index task which was obtained from the Swiss Centre for Affective Science https://www.unige.ch/cisa/emotional-competence/home/research-tools/eri/ available on Qualtrics).

Screening: Piloting indicated that slow internet speeds, poor quality cameras, and failure to adhere to instructions ensuring facial visibility initially resulted in a large quantity of inadequate quality video clips. As such we included a screening study to ensure high-quality videos. Participants were first asked if they had a high-quality webcam and good internet speed to complete all parts of the study that day and the next day. They then read the participant information and completed the consent form, and completed the task recording Video 1 – the hobbies video clip. A research assistant immediately checked their video clip to assess its quality. If the video appeared to be of adequate quality, the participant received a link via Prolific to complete the main study right away. Those with subjectively inadequate video quality or adherence to task instructions could not proceed but were compensated 75p.

Table SI2

Countries of residence of participants in dataset 5

**Country**	**N**
United Kingdom	109
South Africa	99
Portugal	33
Poland	25
Canada	16
Netherlands	15
Italy	12
Greece	11
Mexico	11
Germany	10
France	7
United States	6
Slovenia	5
Spain	5
Sweden	5
Austria	3
Estonia	3
Latvia	3
Chile	2
Hungary	2
Ireland	2
Israel	2
Japan	2
Australia	1
Belgium	1
Czech Republic	1
New Zealand	1

The main study consisted of a battery of social and cognitive measures. To reduce participant fatigue, these were divided into 2 parts (each taking approximately 30 minutes each), and the tasks and questionnaires were presented in an interspersed non-randomised order to introduce variety. After the screening, if participants completed Part 1, they received a link for Part 2 the next day. The majority of participants completed Part 2 within a day of receiving the link. participants completed Part 1, and Part 2 was typically completed the day after but within a maximum of 1 week later. For those that did not, they received daily reminders for 1 week, after which they received partial compensation (£4) and could not continue. The tasks and questionnaires were included to address multiple research questions; the predictions outlined in the current manuscript, and other unrelated questions.

Questionnaires and Tasks in order of completion

Screening:
- Participant Information and Consent
- Video 1 – Hobbies

Part 1:
- Social Network Index Scale (4)
- Social Network Quality Scale (7)
- Social Face Test: a novel task in which naturalistic social interaction video clips are shown and participants are asked to interpret the actors' intentions.

- Social Circles Scale (8)
- Facial Production Task: a novel task in which participant are shown moving images of facial movements and asked to produce them while being video recorded.
- Facial Expressivity Scale: a novel 9-item self-report questionnaire measuring facial expressivity
- Berkeley Emotional Expressivity Scale (9)
- Submissive Behaviour Scale (10)
- Sustained Attention to Response Task (11)

Part 2:
- Video 2 - Scripted video
- Friendship Satisfaction Scale (12)
- Fundamental Social Motives Scale Questionnaire (13)
- Cooperation Game (14)
- Trust Game (15)
- Trust Questions: three trust-related self-report questions from Ben-Ner & Halldorssen (16)
- Video 3 – Partner-directed
- Big Five Inventory Scale (17)
- Social Niche Scale: a brief (8-item) novel questionnaire designed to measure preference for and time spent in cooperative and competitive social environments.
- Social Comparison Scale (18)
- Emotional Recognition Index – Facial Expression Task only (6)

Methods SI3 Social Network Index – modifications

The social network index (4) was modified slightly from its original version to modernise it in datasets 1, 2, 4 and 5. In the original version, participants are asked to indicate how many social contacts within each relationship category they "see or talk to on the phone at least once in a usual 2 week period". While participants might assume this includes virtual contact, we explicitly appended this: "including phone calls, video chat or instant messaging". In relation to the romantic partners question, we removed responses that distinguished between married and unmarried romantic partners, separated, divorced and widowed, as these was unrelated to the social network metrics. Participants could instead indicate "Currently living with a romantic partner", "In a romantic relationship but living apart" and "Single (not married or in a romantic relationship)". Datasets 4 and 5 also included the option to respond "In a romantic relationship with more than one person" and to indicate the number of romantic partners. Finally, datasets 4 and 5 also allowed participants to indicate whether they had step-parents they were in regular contact with, and included step-parents when asking about their romantic partners' parents.

These resulted in minor differences; approximately 1% of participants from datasets 4 and 5 indicated they had more than one romantic partner, and 10% indicated they had a step-parent they were in regular contact with. Together these amounted to approximately 1% of participants' total social networks in datasets 4 and 5, and in the other datasets, many participants would likely have accounted for any step-parents in the 'parents' or 'other relatives' categories. While minor, these differences are controlled for in the models by including dataset as a random effect.

Supplemental References

1. E. Kavanagh, J. Whitehouse, B. M. Waller, Being facially expressive is socially advantageous. *Sci Rep* **14**, 12798 (2024).

2. W. R. Revelle, psych: Procedures for Personality and Psychological Research. (2017).

3. A. Reece, *et al.*, The CANDOR corpus: Insights from a large multimodal dataset of naturalistic conversation. *Science Advances* **9**, eadf3197 (2023).

4. S. Cohen, W. J. Doyle, D. P. Skoner, B. S. Rabin, J. M. Gwaltney Jr, Social Ties and Susceptibility to the Common Cold. *JAMA* **277**, 1940–1944 (1997).

5. J. J. Gross, O. P. John, Facets of emotional Expressivity: Three self-report factors and their correlates. *Personality and Individual Differences* **19**, 555–568 (1995).

6. K. R. Scherer, U. Scherer, Assessing the Ability to Recognize Facial and Vocal Expressions of Emotion: Construction and Validation of the Emotion Recognition Index. *J Nonverbal Behav* **35**, 305–326 (2011).

7. L. E. Flores Jr, J. M. Cyranowski, M. Amole, H. A. Swartz, Prospective assessment of social network quality among depressed mothers treated with brief psychotherapy: The Social Network Quality (SNQ) scales. *Comprehensive psychiatry* **78**, 98–106 (2017).

8. R. I. M. Dunbar, M. Spoors, Social networks, support cliques, and kinship. *Human Nature* **6**, 273–290 (1995).

9. J. J. Gross, O. P. John, Revealing feelings: Facets of emotional expressivity in self-reports, peer ratings, and behavior. *Journal of Personality and Social Psychology* **72**, 435–448 (1997).

10. P. Gilbert, S. Allan, Assertiveness, submissive behaviour and social comparison. *British Journal of Clinical Psychology* **33**, 295–306 (1994).

11. I. H. Robertson, T. Manly, J. Andrade, B. T. Baddeley, J. Yiend, Oops!': performance correlates of everyday attentional failures in traumatic brain injured and normal subjects. *Neuropsychologia* **35**, 747–758 (1997).

12. V. A. Kaufman, J. C. Perez, S. P. Reise, T. N. Bradbury, B. R. Karney, Friendship Network Satisfaction: A multifaceted construct scored as a unidimensional scale. *Journal of Social and Personal Relationships* **39**, 325–346 (2022).

13. R. Neel, D. T. Kenrick, A. E. White, S. L. Neuberg, Individual differences in fundamental social motives. *Journal of Personality and Social Psychology* **110**, 887–907 (2016).

14. D. Kahneman, J. L. Knetsch, R. Thaler, Fairness as a constraint on profit seeking: Entitlements in the market. *The American economic review* 728–741 (1986).

15. B. McEvily, J. R. Radzevick, R. A. Weber, Whom do you distrust and how much does it cost? An experiment on the measurement of trust. *Games and Economic Behavior* **74**, 285–298 (2012).

16. A. Ben-Ner, F. Halldorsson, Trusting and trustworthiness: What are they, how to measure them, and what affects them. *Journal of Economic Psychology* **31**, 64–79 (2010).

17. O. P. John, E. M. Donahue, R. L. Kentle, Big five inventory. *Journal of Personality and Social Psychology* (1991).

18. S. Allan, P. Gilbert, A social comparison scale: Psychometric properties and relationship to psychopathology. *Personality and Individual Differences* **19**, 293–299 (1995).
